# Identifying Corridors among Large Protected Areas in the United States

**DOI:** 10.1371/journal.pone.0154223

**Published:** 2016-04-22

**Authors:** R. Travis Belote, Matthew S. Dietz, Brad H. McRae, David M. Theobald, Meredith L. McClure, G. Hugh Irwin, Peter S. McKinley, Josh A. Gage, Gregory H. Aplet

**Affiliations:** 1 The Wilderness Society, Bozeman, Montana, United States of America; 2 The Wilderness Society, San Francisco, California, United States of America; 3 The Nature Conservancy, Seattle, Washington, United States of America; 4 Conservation Science Partners, Inc., Ft. Collins, Colorado, United States of America; 5 Center for Large Landscape Conservation, Bozeman, Montana, United States of America; 6 The Wilderness Society, Black Mountain, North Carolina, United States of America; 7 The Wilderness Society, Hallowell, Maine, United States of America; 8 Gage Cartographics, Bozeman, Montana, United States of America; 9 The Wilderness Society, Denver, Colorado, United States of America; Clemson University, UNITED STATES

## Abstract

Conservation scientists emphasize the importance of maintaining a connected network of protected areas to prevent ecosystems and populations from becoming isolated, reduce the risk of extinction, and ultimately sustain biodiversity. Keeping protected areas connected in a network is increasingly recognized as a conservation priority in the current era of rapid climate change. Models that identify suitable linkages between core areas have been used to prioritize potentially important corridors for maintaining functional connectivity. Here, we identify the most “natural” (i.e., least human-modified) corridors between large protected areas in the contiguous Unites States. We aggregated results from multiple connectivity models to develop a composite map of corridors reflecting agreement of models run under different assumptions about how human modification of land may influence connectivity. To identify which land units are most important for sustaining structural connectivity, we used the composite map of corridors to evaluate connectivity priorities in two ways: (1) among land units outside of our pool of large core protected areas and (2) among units administratively protected as Inventoried Roadless (IRAs) or Wilderness Study Areas (WSAs). Corridor values varied substantially among classes of “unprotected” non-core land units, and land units of high connectivity value and priority represent diverse ownerships and existing levels of protections. We provide a ranking of IRAs and WSAs that should be prioritized for additional protection to maintain minimal human modification. Our results provide a coarse-scale assessment of connectivity priorities for maintaining a connected network of protected areas.

## Introduction

Protected areas or ecological reserves (e.g., wilderness areas, national parks) form the foundation of conservation strategies to sustain biological diversity [1]. Established to reduce human impacts, protected areas are intended to maintain populations of species and ecological functions [2, 3]. Isolated protected areas, however, may not provide for species migration and dispersal or ecological flows of materials required to sustain genetic and species diversity, population recovery, and ecosystem processes [4]. Protected areas unconnected to a network may serve only as temporary insular ecosystems, vulnerable to population isolation or environmental change [5, 6] and may be at greater risk of experiencing local species extirpations.

A long history of ecological and conservation science has addressed questions of reserve design, extinction risks from isolation, and the value of connectivity [7]. Moreover, creating, restoring, and maintaining large, connected networks of protected areas has emerged as one of the highest priorities for conservation in the age of climate change [8–11]. Providing organisms opportunities to move long distances, possibly over many generations, is a necessary conservation strategy to ensure that species can shift their distributions to track expected climate change [12]. Maintaining relatively natural and undeveloped connections between protected areas in a network of ecological reserves may be the best means for conserving biodiversity now and in the future [13].

In response to a growing number of theoretical frameworks [14–16] and empirical demonstrations of the importance of connectivity for maintaining biodiversity [17–19], a suite of analytical tools has been developed to identify linkages or potential corridors between protected areas, known populations, or potential habitat [20,21]. Two primary approaches to assess connectivity have been used [22]: those based on species-specific habitat requirements and those focused on identifying corridors using landscape “naturalness” as a proxy for the needs of multiple species [23,24]. Most connectivity models assume that organisms moving across a landscape or region will incur costs of movement (or resistance to travel) corresponding to energetic expense or risk of mortality [25]. Many connectivity models thus identify corridors where cost of, or resistance to, movement is minimized [26,27].

Connectivity models based on a landscape’s “naturalness” (i.e., degree of human modification) likely represent well the potential costs of movement, especially for species that are sensitive to human disturbance [24]. In fact, many species-specific connectivity models assume animal avoidance of human-altered areas [25]. As climate change forces shifts in the distribution of species and habitats [12,28], identifying large, continental-scale corridors of high naturalness (*sensu* [23]) may also be a critical conservation strategy to sustain biodiversity [3].

Many federally-owned and -managed lands of the United States (e.g., lands managed by the U.S. Forest Service and Bureau of Land Management) are currently undergoing land-use planning. Identifying regionally important corridors that may provide ecological connections between protected areas, as well as federal land units that fall along these corridors, can inform decisions regarding additional land protection or mitigating impacts of land use, including resource extraction, motorized recreation, and other activities. In some cases, land units are being considered as candidates for elevated levels of protection. For example, Inventoried Roadless and Wilderness Study Areas could be recommended for permanent protection, or alternatively for “release” to increased recreational or commercial use. Quantifying the contribution of these candidate land units for maintaining a connected network of large protected areas may help the public and land managers prioritize which units should receive elevated levels of protection.

Here, we develop a national-scale connectivity model with the aim of identifying potential priorities for maintaining connectivity among existing, large protected areas of the contiguous United States of America (hereafter, U.S.). This differs from a previous national analysis of connectivity developed by Theobald et al. [23] that identified pathways through lands with low human modification, but without an explicit aim to model potential connections between protected areas. Here, we identify the least human-modified corridors between large protected areas based on two different indices of human modification (or inversely, naturalness) to create a composite map of corridors that highlights agreement among models run with various assumptions about how connectivity potential may be influenced by the degree of human modification of lands. We used the composite corridor map to ask two questions about connectivity between existing protected areas. First, we asked which conservation lands not within our pool of identified large protected core areas contribute to the identified corridors. Second, we asked which Inventoried Roadless Areas and Wilderness Study Areas on U.S. federal lands would contribute most to connectivity if added to the protected area system in the future.

## Materials and Methods

### Overview of Approach

Connectivity modeling requires numerous decisions and assumptions resulting in myriad combinations of factors to consider [29], each of which can affect model results. We used Linkage Mapper [30], which, like other connectivity modeling tools (e.g., Circuitscape, [31]), requires a set of core areas to connect and a “resistance surface” (i.e., a mapped index reflecting cost or risk of movement) as inputs. Linkage Mapper is well-suited to identify corridors between protected areas, and produces a raster surface of cost-weighted distances normalized by least-cost paths between cores. This allows users to evaluate and prioritize land-unit polygons based on their mean corridor value. We found that at the scale with which we were working (the U.S.), Linkage Mapper required less processing time than alternatives such as Circuitscape. Here, we developed connectivity models using a relatively simple rule-set to define our core areas. To minimize the potentially arbitrary effect of selecting a single resistance surface, we produced our connectivity map as a composite of model outputs employing four different resistance surfaces representing gradients in human-modification of lands (e.g., from roads or agricultural land conversion).

### Identification of Core Areas

There are numerous ways to define protected areas; for our analysis we chose to focus on large protected areas with conservation mandates requiring maintenance of biodiversity and prevention of land conversion, commercial resource extraction, and intensive motorized recreation. We focused on “large” highly-protected core areas because these areas likely serve as important regional or national conservation lands hosting the greatest number of species, largest populations, and higher probability for maintaining intact ecological processes (e.g., relatively unaltered disturbance regimes) [3].

**Fig 1:**
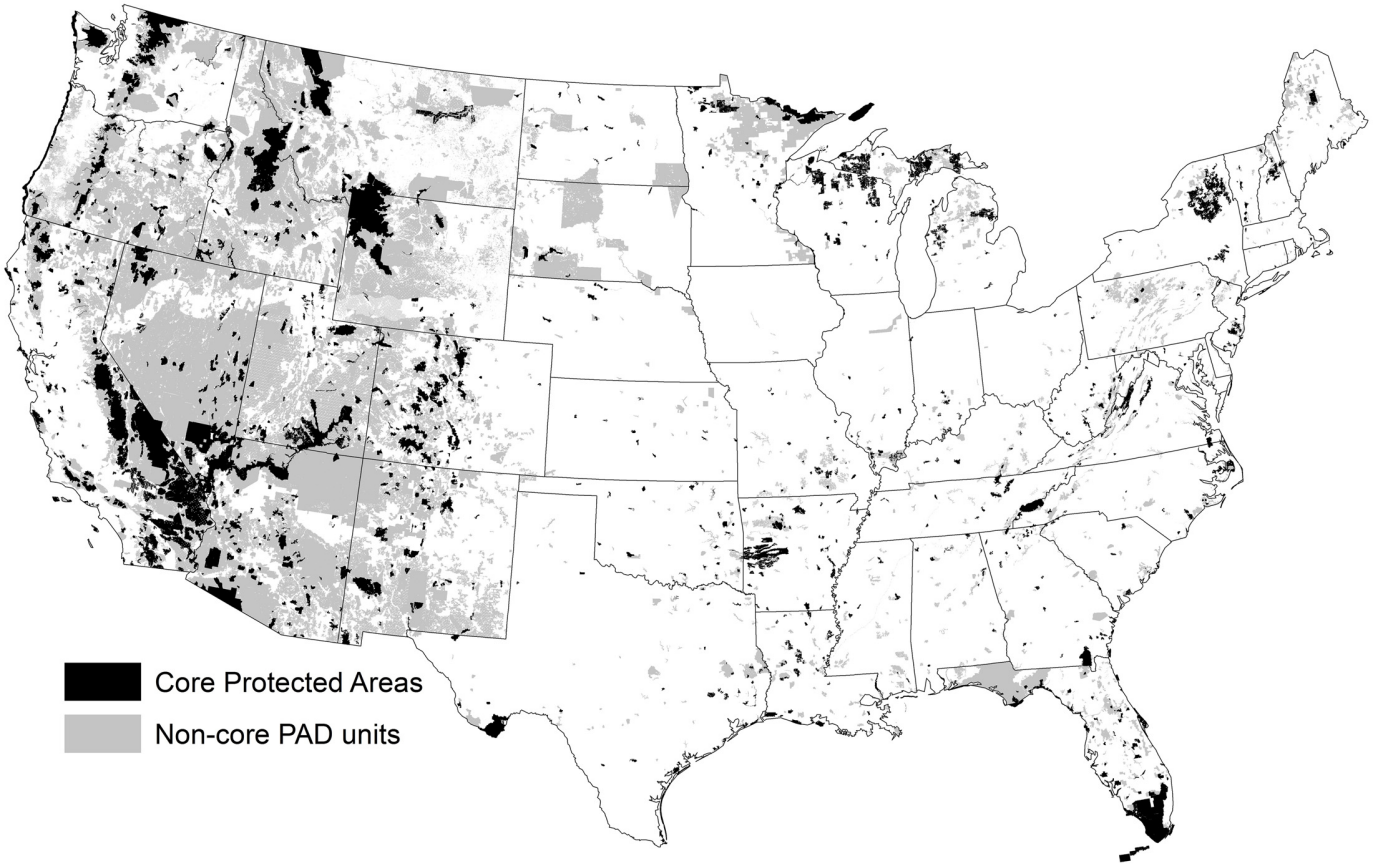
Map of the contiguous United States showing large core protected areas and Protected Areas Database of the U.S. units not included in our definition of core protected areas.

To define and identify our protected cores, we used a simple ruleset. First, we selected all polygons from the National Wilderness Preservation System (NWPS) database (Wilderness Institute 2014), irrespective of size, as these are the most highly-protected lands in the U.S., and because even small wilderness areas represent important conservation lands in some regions [32]. Although other small non-wilderness lands may also serve as important regional protected cores, wilderness areas are designated and managed under federal legislation providing nationally-consistent guidance on management. Second, we selected areas mapped in the Protected Area Database (PAD) v 1.3 [33] with GAP 1 or 2 status, as these lands include legislative or management direction to maintain biodiversity, limit commercial resource extraction, and prohibit land cover conversion. Third, we selected all GAP 3 status lands managed by the National Park Service (NPS) that are not classified as “cultural” parks (e.g., military parks), so as to include all “natural” NPS units. Because most “natural” NPS units are listed as GAP 1 and 2, this step added only one unit in Tennessee. Fourth, we merged adjacent protected areas into one larger contiguous core so that protected areas that share a border were counted as only one area (e.g., Yellowstone National Park and the adjacent Absaroka-Beartooth Wilderness Area are counted as one large core). Fifth, we eliminated core area polygons <4,046 hectares (10,000 acres) to limit our analysis (with the exception of some stand-alone wilderness areas) to connections between “large” blocks of highly protected core areas. Sixth, one large core protected area (Dakota Tallgrass Prairie Wildlife Management Area) was removed from our pool of protected cores because much of its area is still privately owned and managed with an agricultural land cover type even though it is listed as GAP 2 status. This ruleset yielded 2,084 protected core areas (Fig 1), which we considered our national cores to be connected. These cores represent various ownerships, but over 82% of the core areas are managed by the four principal federal land agencies: U.S. Forest Service (33.9%), National Park Service (23.8%), Bureau of Land Management (14.5%), and Fish and Wildlife Service (10.1%).

### Creating Resistance Surfaces

To model corridors among our selected pool of protected core areas, we developed resistance surfaces based on two different efforts to quantify and map anthropogenic alterations to ecosystems: Aplet et al.’s wildness index [34] and Theobald’s map of human modification [35] (see below for more detail). Resistance surfaces represent relative cost (e.g., energetic expense or risk of mortality) of passing through a gridded mapped surface and can be used to calculate cost-weighted distance (CWD) away from core areas. CWD values are used to produce least cost corridors and least cost paths (LCP). In Linkage Mapper, least cost corridor values are produced by summing CWD away from two cores, say core A and core B. The CWD_AB_ sum is then normalized by LCP values between cores A and B (LCP_AB_) to produce normalized least cost corridor (NLCC_AB_) values (i.e., NLCC_AB_ = CWD_A_ + CWD_B_−LCP_AB_) so that grid cells along the LCP equals 0. NLCCs for all corridors are then mosaicked together to produce a network of corridors representing cost distance (lower values have better potential to serve as a corridor).

In our national assessment of corridors between our identified pool of protected areas, we assumed that organisms will selectively or more easily (i.e., with lower mortality risk) move through the wildest and most natural lands. We used the maps of wildness and human modification to identify corridors that minimized anthropogenic disturbance of corridors. The wildness index and map of human modification each incorporate spatial data layers on land cover, distance and impacts from roads, hydrological impairment, degree of light pollution, and other indicators of the degree to which land is modified by humans and maintains relative ecological integrity. However, the models differ in several of their inputs and the way those inputs are manipulated to characterize naturalness and human impact. The wildness index [34] was updated with data reflecting conditions circa 2008 and is mapped at a resolution of 1 km^2^. It is based on a conceptual framework [36] that considers human control of ecological processes and the ecological condition of lands and consists of ordinal data reclassified and summed. Human modification [35] uses a “fuzzy algebraic sum” of mapped data on stressors of ecological composition, structure, and function to create a ratio from 0 to 1.0 from 30 m data, aggregated to 90 m or coarser resolutions. These measures of naturalness are highly correlated (r = 0.83), but because they were developed for different purposes, using different methods, we used each to identify areas of agreement between corridors identified through different models (see below).

**Fig 2:**
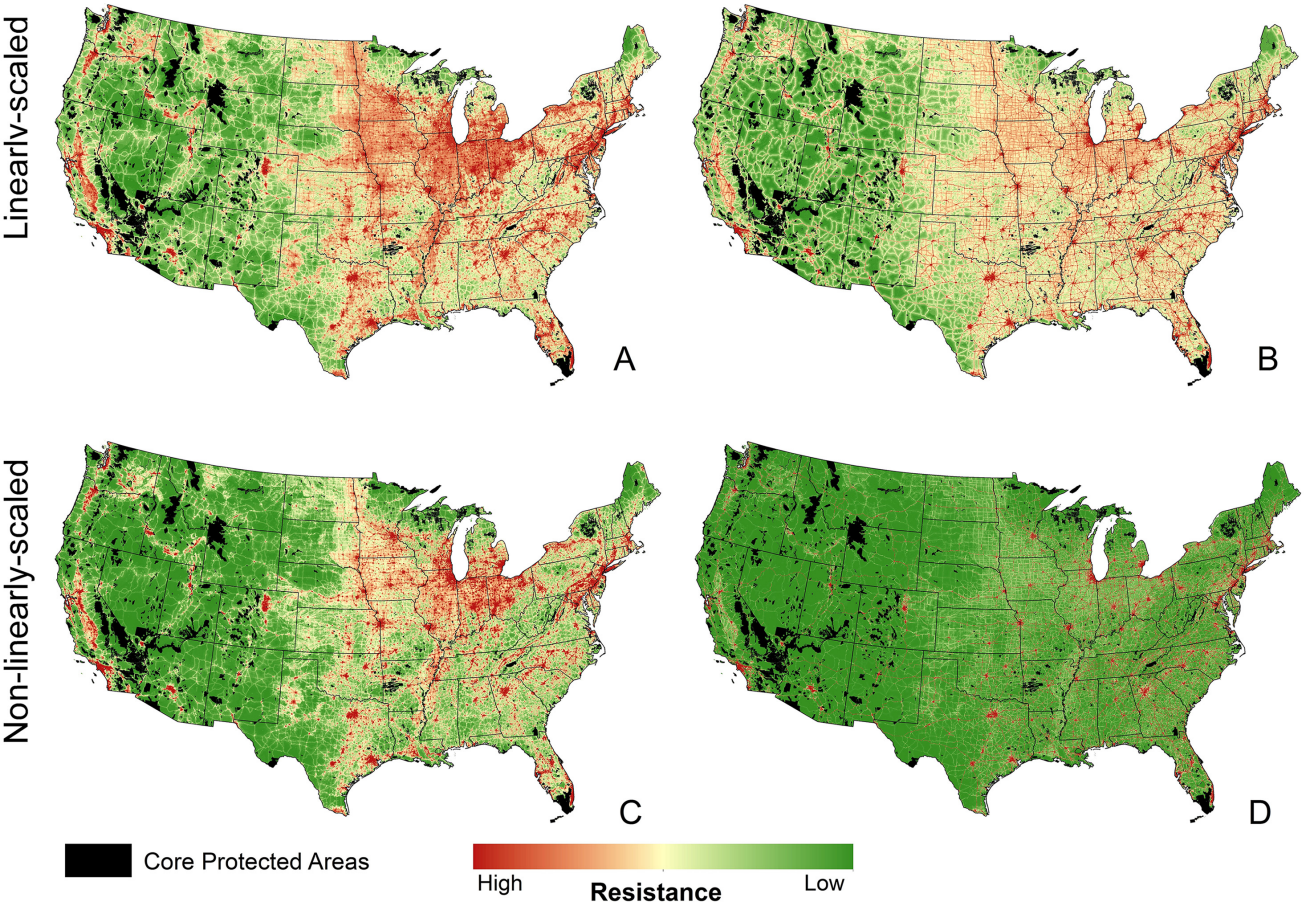
Linear (top) and non-linear (bottom) versions of resistance surfaces based on wildness (left) and human modification (right) used to model connectivity between large protected areas. See S1 Fig for distribution of resistance surfaces in relation to original indices.

While connectivity models based on naturalness perform well when compared against species-specific habitat connectivity models [24], the degree to which movement risk, animal avoidance, or ecological condition changes along gradients of wildness or human modification is mostly unknown. Therefore, we created four resistance surfaces by scaling each map of wildness and human modification (HM) two different ways allowing us to create a composite corridor map that incorporates alternative methods for converting indices of naturalness into resistance surfaces for corridor identification [16,37]. Specifically, we rescaled wildness and HM to represent both linear and non-linear gradients of landscape resistance. For example, a one unit decrease in wildness or HM caused by moderate anthropogenic impact to an ecosystem may result in either a one unit change in resistance or greater than one unit change in resistance to movement, risk of mortality, or behavioral avoidance (e.g. [38], S1 Fig). For linear scaling of wildness, we assigned resistance values ranging from 1 (most wild) to 25 (most developed) (S1A Fig, Fig 2A). For non-linear scaling we squared this surface to produce a resistance surface that ranged from 1 to 625 (S1A Fig, Fig 2B). To produce the resistance surfaces based on HM, we first aggregated the data to a 1 km resolution to reduce processing time using bilinear interpolation of a 270-m resolution gridded layer. We then transformed the original index so that the linearly-scaled resistance surface based on human modification ranged from 1 to 1001 using (HM×1000)+1 (S1B Fig, Fig 2C). For the non-linearly scaled resistance surface based on human modification, we scaled the HMI by a power of 10 using (HM+1)^10^ (S1B Fig, Fig 2D). We assumed that relative resistance to movement from intensive agricultural to suburban to urban areas would continue to increase along this gradient of anthropogenic impact [38]; therefore we did not model connectivity with resistance surfaces that saturate (i.e., reach an asymptote) along the wildness index or human modification.

Modeling corridors using this combination of resistance surfaces allow us to compare corridor outputs based on linearly-scaled wildness and HM, as well the effects of the different scaling methods on cost-weighted distances based on wildness and HM (S2 Fig). Here we were more interested in running the national connectivity models based on various assumptions of resistance for further use in a composite corridor map that combines all outputs (see below).

### Connectivity Model

We modeled connectivity using each of the four resistance surfaces (Fig 2) and their derived cost-weighted distances (S2 Fig). Linkage Mapper allows users to limit identified corridors based on maximum geographic distance, cost-weighted distance, and on other criteria. We first ran the connectivity model using each of the four resistance surfaces with no limitations on geographic or cost-weighted distances. The resulting model identified corridors between protected areas separated by large distances (>500 km) and highly altered lands (e.g., urban areas); see S3 Fig. To avoid inclusion of these unrealistic corridors, we reran each model, but limited the maximum geographic distance between the edges of cores to be connected to 300 km, based on maximum-dispersal distance data from wide ranging mammals [39]. We then evaluated the cost-weighted paths using the values of each least cost path from this output (S4 Fig). We decided to remove the 10% most costly paths (red paths in S4 Fig) to further limit the corridors between protected areas. This step removed unrealistic corridors that passed through highly altered lands. Limiting the corridors based on these maximum geographic and cost-weighted distances resulted in more conservative corridor priorities that link core areas separated by reasonable and biologically-informed distances (i.e., we assume connecting core areas > 300 km apart is much less likely to reflect movements of terrestrial species, processes, or materials). For all model runs, corridors between protected areas were discarded if the least-cost paths intersected an intermediate core area [30]. We did not refine the network using other options available in Linkage Mapper (e.g., we did not limit the number of connected neighboring cores).

### Producing a Composite Corridor Map

**Fig 3:**
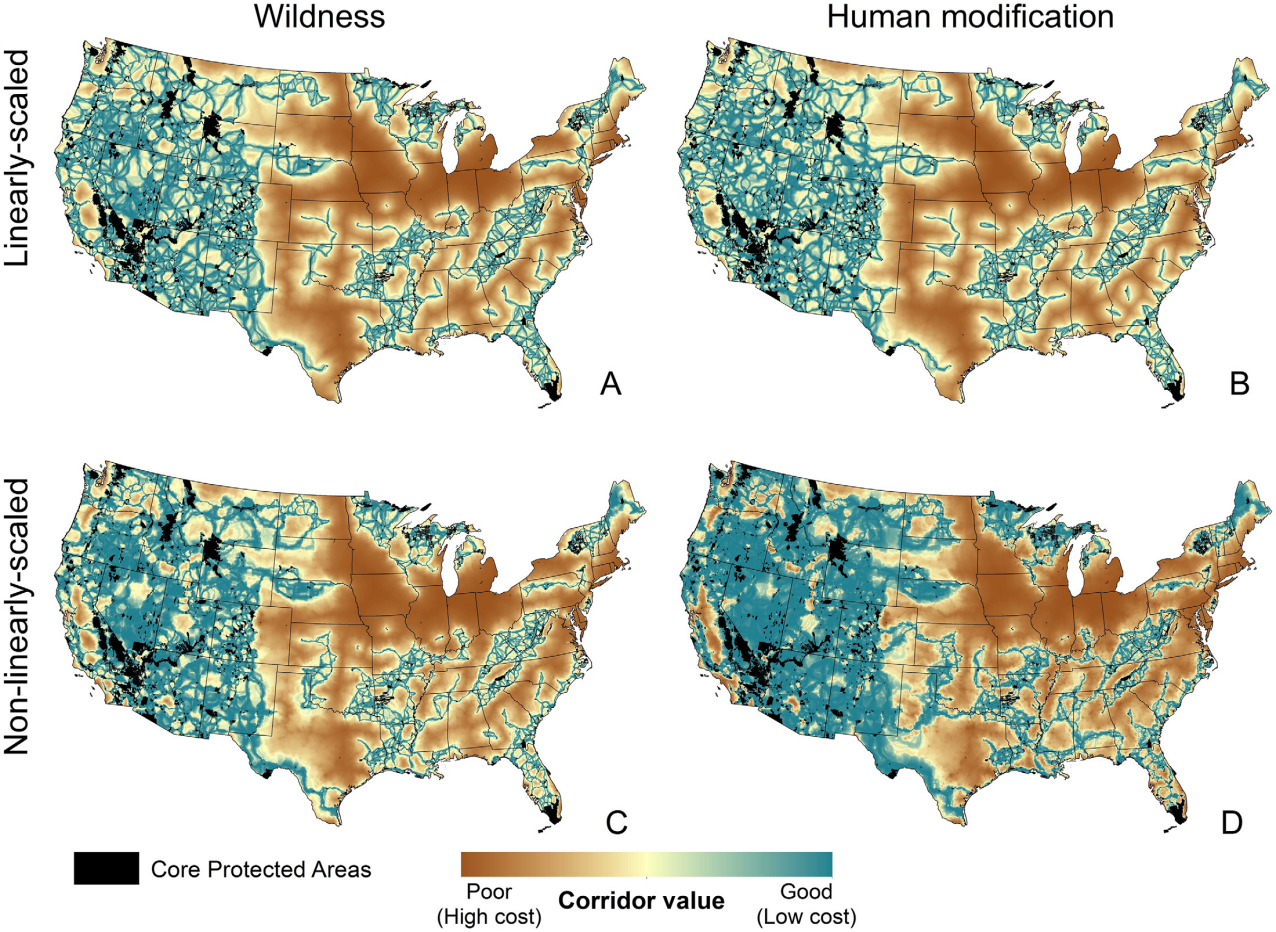
Normalized least cost corridors (after removing the linkages >300 km shown in S3 Fig and the 10% worst linkages shown in S4 Fig) from Linkage Mapper connectivity models based on linear (left) and non-linear (right) versions of resistance surfaces using wildness (top) and human modification (bottom).

After producing the four conservative corridor maps (Fig 3), we created a composite map by combining each output to identify areas with high agreement among the four outputs. To do this, we first reclassified the corridor values of each output into deciles and assigned the lowest cost decile a value of 10, the next lowest decile a value of 9, etc., so that lower cost linkages received higher corridor values. We reclassified each corridor value map in this way and then summed these new reclassified maps so that the composite map ranged from 4, where all of the highest cost (i.e., worst) corridor values overlapped to 40, where all of the lowest cost (i.e., best) corridors of each output overlapped. Using the sum of reclassified deciles of our output produced qualitatively similar spatial patterns as the mean of the cost-weighted distance corridor outputs. However, we produced our composite map using the sum of reclassified deciles because the numerical values (ranging from 4 to 40) were easier to interpret than the average cost-distance corridor values.

### Mean Composite Corridor Values of Management Areas

We calculated the mean composite corridor value for land management units not included in our pool of large protected core areas. Specifically, we calculated the mean composite corridor value of PAD land management units (N = 4,360 areas) falling outside our core areas (“non-PAD core units”; grey polygons in Fig 1). We also evaluated how mean composite corridor values were distributed among land units with different GAP status values and agencies. This analysis included smaller (<4,046 ha) GAP 1 and 2 management units excluded from our large core protected areas.

Finally, we evaluated the mean corridor value of some federal land units that may be candidates for elevated protection status but that are not currently legislatively protected as wilderness or national park. Specifically, we calculated the mean composite corridor value of all U.S. Forest Service Inventoried Roadless Areas and Wilderness Study Areas (N = 1,148) and BLM Wilderness Study Areas (N = 268) to identify potential land units important for maintaining a national network of large, connected, protected areas and that are potential candidates for elevated levels of protection (*sensu* [40]). We excluded units ≤ 3,000 ha from this analysis to ensure an adequate sample of at least 30 grid cells from the composite corridor value raster.

## Results

Use of the four resistance surfaces (Fig 2) resulted in some differences among the cost-weighted distances (S2 Fig), least-cost paths (S4 Fig), and least-cost corridor maps (Fig 3). All continuous raster maps shown here are displayed using the “histogram equalize” stretch method. Maps resulting from linearly-scaled wildness and HM were very similar, but corridors produced using non-linearly scaled resistance surfaces tended to have large areas where low-cost corridors were less distinct based on the scaling of the data (e.g., Central and Northern Basin and Range ecoregions in Nevada and Oregon). The large, contiguous patches of high connectivity values reflect large patches of relatively wild and unaltered lands with low resistance values. The linearly-scaled resistance surfaces tended to produce narrower corridors.

**Fig 4:**
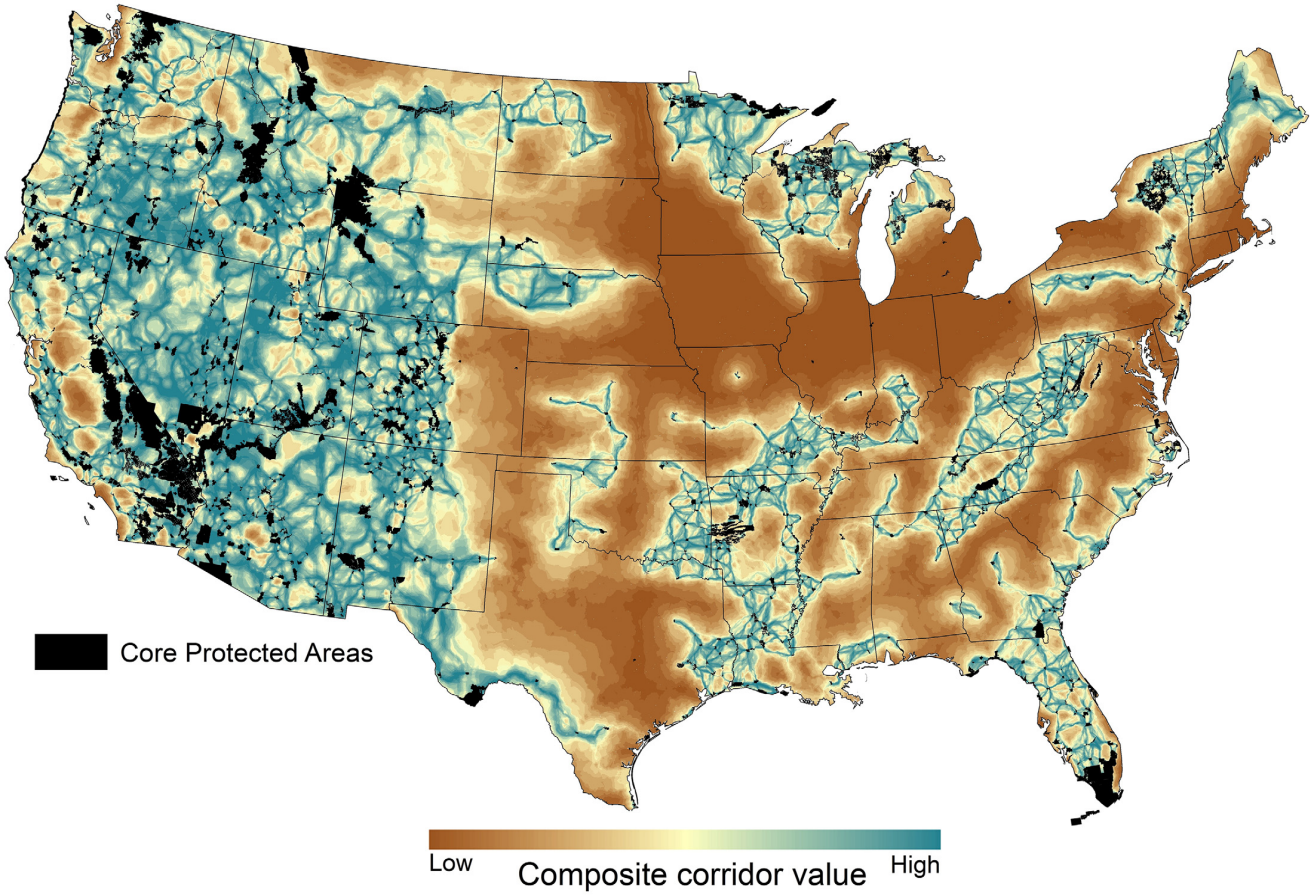
Composite corridor value between large protected core areas, which was calculated by summing and reclassifying normalized least-cost corridors shown in Fig 3. Black polygons are large core protected areas used in our analysis. Raster data are available as S1 File.

Despite these differences, all four maps exhibit coarse-scale similarities (Fig 3), and the composite corridor map (Fig 4) quantifies broad areas of agreement among least-cost corridors (available as S1 File). Western regions tended to have lower resistance values leading to higher corridor values, but many eastern regions also host relatively well-connected networks of protected areas (Figs 3 and 4). In particular, the southern Appalachians, northern Lake States, Ozarks, northern New England, and Florida each contain a connected network of protected areas. In addition, the upper Missouri River basin, Black Hills/Sandhills region, and the Texas border region all show the potential for functional connectivity to the West. Smaller regions of connectivity are apparent in the east Texas Piney Woods, the northern Allegheny Plateau, and the Carolina coast.

**Fig 5:**
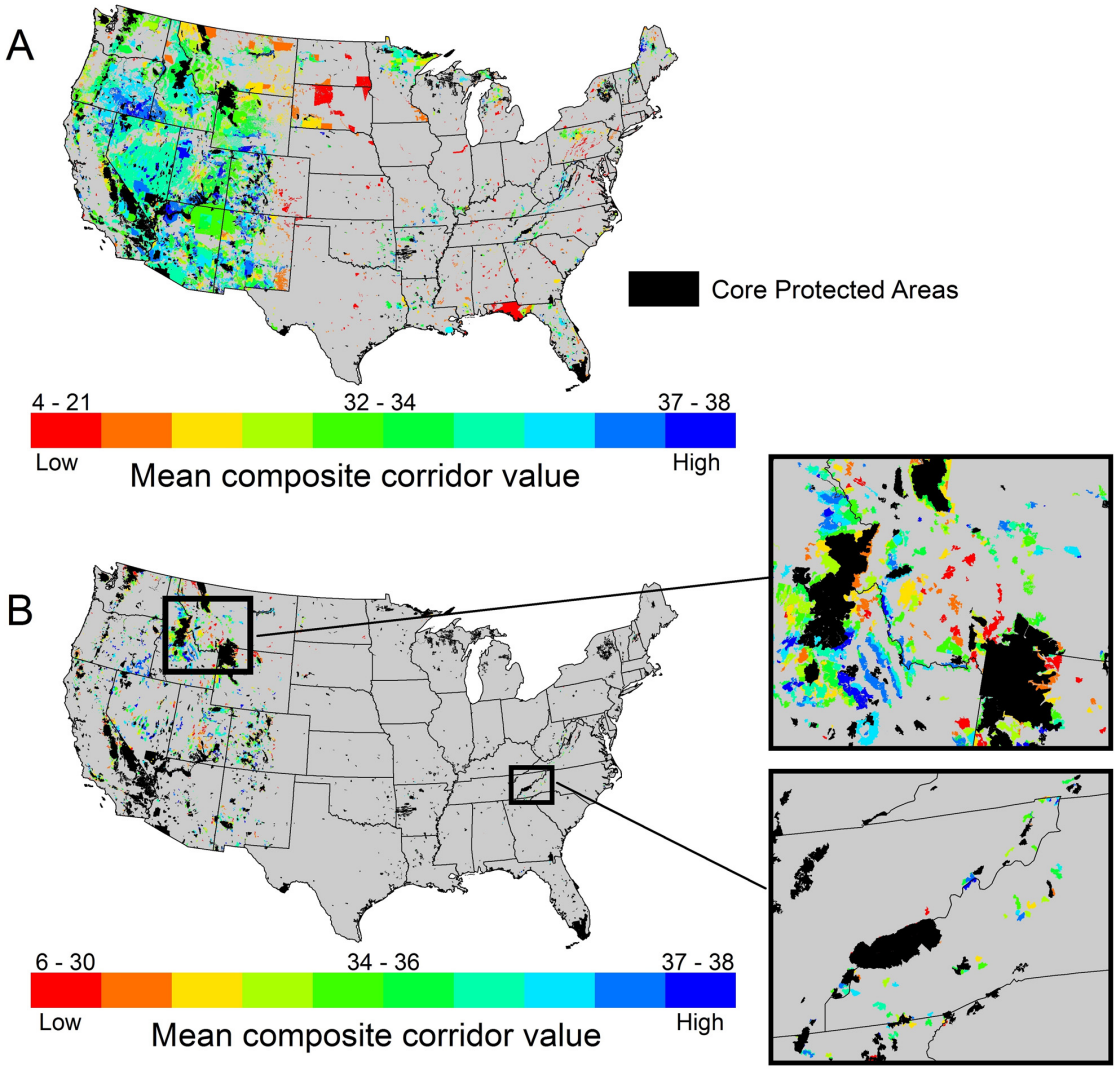
Mean composite corridor values (classified into deciles) among non-core Protected Area Database land units (A) and federal inventoried roadless and wilderness study areas (B). Roadless and wilderness study areas are shown in greater detail for the Northern Rockies and Southern Appalachian Mountains.

Some PAD units outside of core protected areas were clearly located along high-value corridors, while others lie far away from large protected cores (Fig 5A). Concentrations of highly connected but unprotected PAD units exist in southeastern Oregon, the Great Basin and Colorado Plateau, western Maine, and the “Idaho High Divide” country between the Frank Church—River of No Return Wilderness and Yellowstone National Park. Although variability was high, mean composite corridor values for GAP status 1 land units (N = 19) tended to be lower (i.e., poorer quality) than Gap 2 (N = 524), Gap 3 (N = 3,113), and Gap 4 units (N = 974; S1 Table). Three land trust units had the highest mean composite corridor value of any class of non-core PAD lands (S1 Table). Of federal agencies, BLM land units had the highest corridor values, followed in descending order by the Department of Energy, U.S. Forest Service, Natural Resource Conservation Service, Bureau of Reclamation, Bureau of Indian Affairs, Tennessee Valley Authority, Department of Defense, Agricultural Research Service, Fish and Wildlife Service (FWS), National Park Service (NPS), and National Oceanic and Atmospheric Administration. Many other non-federal agency lands (e.g., lands managed by states) had higher mean corridor values than federal land managed by FWS and NPS (S1 Table).

The composite corridor value also facilitates assessment of the relative connectivity value of all 2,876 IRAs and WSAs on national forests and BLM lands (Fig 5B; S2 File). The mean composite corridor value for U.S. Forest Service IRAs and WSAs and BLM WSAs (33.8) was greater than the overall average of non-core PAD lands (31.2). BLM WSA lands had a higher average corridor value (34.6) than USFS IRAs (33.6) and USFS WSAs (34.0). S2 File ranks all USFS and BLM IRAs and WSAs according to their mean composite corridor value.

## Discussion

Corridors identified here represent the “wildest” or most “natural” lands that may provide broad-scale ecological linkages between large protected core areas. Maintaining or enhancing the relatively high degree of naturalness and low degree of human impact along these corridors may be an important strategy to ensure that the large protected areas in the U.S. do not become isolated and are maintained in a connected network [3]. Such continental-scale connectivity is a critical component to conserving ecosystems and biodiversity under a changing climate and accelerated land use change [35,41]. Maintaining relatively natural corridors between protected areas should allow mobile organisms short-term opportunities for movement, while providing more sessile organisms opportunities to move over generations.

Our connectivity assessment could be used in regional conservation strategies, administrative planning, or legislative or executive efforts (such as wilderness bills or national monument proclamations) that support important linkages between protected areas. Corridors identified here may be among the most important areas on which to focus conservation efforts, including the elevation of their protective status through conservation designations (e.g., permanently limit human impacts of an area by increasing the level of land protection from an inventoried roadless area to recommended or legislated wilderness). We demonstrate one relatively easy way to evaluate connectivity priorities by calculating the mean composite corridor value of different ecoregions and land management units. Here, we focused on lands ≥ 3,000 ha within the PAD, IRAs, and WSAs, but similar methods could be used to evaluate individual national forests or BLM jurisdictions to help prioritize areas to maintain connectivity (e.g., [42]). Prioritizing lands to maintain connectivity could result in management objectives that maintain natural land cover and limit development along the best corridors. It can also help inform designation to higher levels of protection on high priority lands (e.g., Basin and Range National Monument). By far the greatest number of PAD units outside of core protected areas are managed by the U.S. Forest Service and Bureau of Land Management, and many of these units (IRAs and WSAs) are eligible to receive a higher level of protection through administrative or legislative means.

Our results demonstrate that some PAD lands outside of existing cores, even those with low levels of protection (Gap 3 and 4 lands), possess high value as corridors and should be considered for inclusion in a national network of large protected areas. Although federal agencies dominated the total land area evaluated in this analysis, many non-federal agency and private lands possessed high connectivity importance. Maintaining connectivity and functioning corridors will require that conservation strategies be developed across all lands, even when their management objectives may be less protective or lack explicit goals to maintain biodiversity. For example, S1 Table shows that lands administered by universities, the NRCS, Regional Agencies, and private landowners all can make important contributions. This assessment could also inform land trust efforts to prioritize acquisition of conservation easements on lands that contribute to broad-scale connectivity. Cross-ownership efforts to create or sustain a connected network of protected areas will be challenging and require further study, including economic cost-benefit analyses coupled with further ecological research (*sensu* [43]).

Although our study focused on identifying linkages among large and highly protected core areas, similar models could be run to investigate corridors between much smaller—but ecologically important—core areas with varying levels of maximum distance thresholds between cores. These analyses may be important supplements to the national-scale analysis presented here for identifying local connectivity priorities. Given the expanse of our study (the U.S.), we chose to focus on identifying corridors between the largest and most-well-protected core areas with maximum distances between cores of 300 km, reflecting maximum dispersal of large wide-ranging mammals [39, and see 24]. However, we acknowledge that different core areas and maximum distances will yield different outcomes. While we developed our corridor maps based on assumption of the widest-ranging species, we did not explicitly consider any species-specific habitat requirements. We assume if the wild or natural character of lands are maintained or restored between protected areas that animals with shorter dispersal distances would be also benefit (see discussion of “umbrella” species in [44]).

At more regional scales, other smaller core areas could be used in similar analyses with varying assumptions (see S5 Fig). Corridor values may change depending on choices in number and location of cores and the maximum distance between cores. In situations where core areas are few and maximum distances used to connect cores is relatively low, corridors may be absent between protected areas. Direct connections between cores can be made by increasing the number of core areas (e.g., by reducing the minimum size threshold or changing assumptions about what is considered a core protected area) so that they fall between large cores and serve as “stepping stones” [45] or by increasing the maximum distances between cores. Users of our national model outputs should be aware of these sensitivities.

Our final maps removed the 10% most costly corridors. We pruned the connections in this way to ensure that the “worst” corridors were not reflected in the final map of connectivity priorities. These relatively developed and human modified corridors may be too far modified to actually function as high value linkages, though our 10% cutoff was arbitrary and the functionality of corridors both above and below this threshold is expected to depend on many factors. S4 Fig shows where these pruned corridors occurred, which was mostly in the agricultural and highly developed Mid-west and eastern U.S. However, we also recognize that these relatively costly linkages may actually be critical regions on which to focus connectivity efforts. For example, corridors passing through the highly altered Tennessee Valley between protected areas on the Cumberland Plateau and the Great Smoky Mountains National Park were removed based on our 10% highest cost pruning but could actually represent important connectivity potential for organisms such as black bears (*Ursa Americana* [46]). While our final maps presented here focus on the best corridors, we believe that even costly linkages could represent important priorities for maintaining or restoring connectivity.

Models that identify landscape permeability irrespective of existing, designated protected cores are an important—and complementary—means of identifying national connectivity priorities (*sensu* [23]). In fact, many corridors our model identified were similar to flowpaths identified by Theobald et al. [23]. Other efforts have emphasized the importance of riparian areas for the value as functional or potential corridors [13,47]. We did not explicitly incorporate riparian features in our models, but our results could be combined with efforts focused on riparian corridors (e.g., [48]). We view the model outputs presented here and other similar efforts [23] as presenting a reasonable first-approximation for identifying lands that should receive a higher degree of protection through management policy, legislation, or executive action that establish and sustain a connected network of large protected areas in the U.S. Such efforts are ongoing through land management planning of federal agencies, investment decisions by land trusts, and legislative actions by policy makers. Our models could be supplemented or modified based on any available data on individual organisms’ dispersal needs or habitat models generated by more in-depth study [24].

## Conclusions

Our results provide an initial identification of priorities to create a national connected network of large protected areas. As federal land agencies begin assessments of connectivity, the models presented here could serve as a coarse-filter assessment for evaluating regional connectivity priorities. They could also serve in the evaluation of individual land units and their potential role in maintaining connectivity among large core protected areas. Land units with high connectivity value could be prioritized for future protection to maintain potential large-scale connectivity. Maintaining such large-scale corridors between protected areas may ensure that protected cores are connected via a system of relatively natural lands. These lands may allow individual wide-ranging animals to disperse via corridors in the short-term [24], with potential for long-term migration and dispersal to take place by organisms with more limited dispersal abilities.

## Supporting Information

S1 Fig Original scaling of the wildness index (A) and human modification (B) in relation to the linear (hashed line) and non-linear (solid line) transformations used to produce resistance surfaces (maps shown in Fig 2). (TIF)

S2 Fig Cost-weighted distance from the protected core areas based on linear (top) and non-linear (bottom) versions of resistance surfaces using wildness (left) and human modification (right). (TIF)

S3 Fig Normalized least-cost corridors from Linkage Mapper connectivity models based on linear (left) and non-linear (right) versions of resistance surfaces using wildness (top) and human modification (bottom). The mapped outputs shown here do not eliminate linkages between protected core areas based on any maximum geographic or cost-weighted distance, and only provided here as a reference of the most liberal modeled corridors.
(TIF)

S4 Fig Least cost paths classified into declines from best (low cost, blue) to the 10% worst (high cost, red) for connectivity models based on linear (left) and non-linear (right) versions of resistance surfaces using wildness (top) and human modification (bottom). (TIF)

S5 Fig Modeled corridors vary depending on the number of cores (black polygons) to be connected, as well as the maximum Euclidean distance between cores. Here, we show a model experiment where we vary the maximum distance from 100 km to 300 km and cross this “treatment” with different number of cores (black polygons). The model runs with “few cores” included all of the large protected cores shown in Fig 1, while the model runs with “many cores” include all cores from Fig 1, plus all roadless and wilderness study area lands. Here, we demonstrate the sensitivity of Linkage Mapper to assumptions of core locations and maximum distances while demonstrating the importance that “stepping stones” may play in connectivity for a 3 state region (Idaho, Wyoming, and Montana). Connectivity that is limited by maximum dispersal distance between core areas may be overcome if additional smaller cores can serve as stepping stones between larger core areas.
(TIF)

S1 File Gridded raster data of the composite corridor value and large core protected areas used in connectivity model presented here. (7Z)

S2 File Table of mean composite corridor values for all Inventoried Roadless Areas or Wilderness Study Areas ≥ 3,000 ha. USFS = US Forest Service; BLM = Bureau of Land Management; FWS = Fish and Wildlife Service; IRA = Inventoried Roadless Area; WSA = Wilderness Study Area.
(CSV)

S1 Table Mean and range of composite corridor values for federal, state, and local management agencies among units with varying GAP status values (1 = most protected; 4 = least protected). The number of units (N) is also reported. Agencies are rank ordered based on their overall mean corridor value so that agencies with the best corridor values are ranked higher.
(DOCX)
