# Supplementary figures and images for: Identifying Corridors among Large Protected Areas in the United States
Source: PLoS One. 2016 Apr 22;11(4):e0154223. doi: 10.1371/journal.pone.0154223 (PMC4841590; doi:10.1371/journal.pone.0154223)

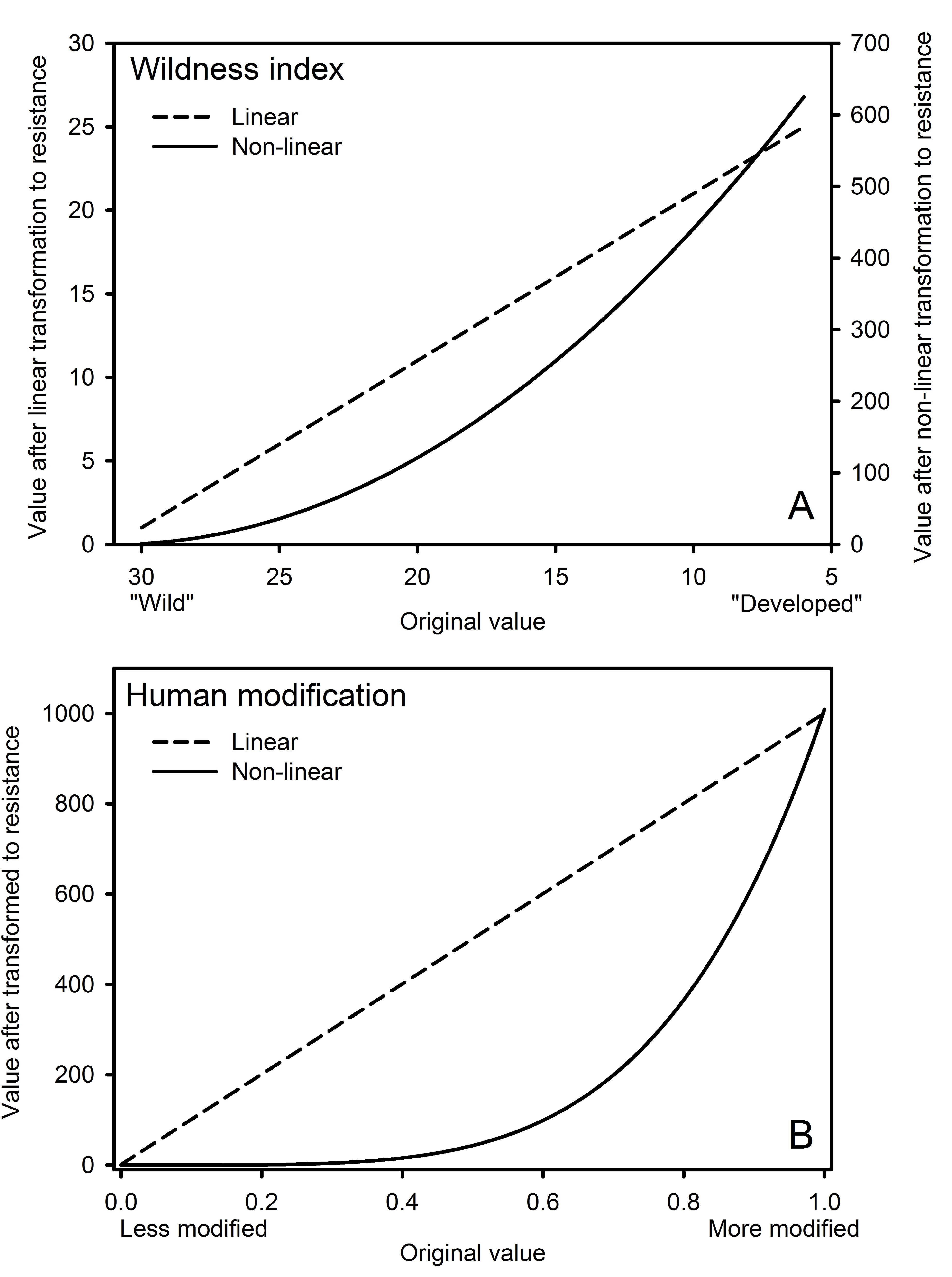

Supplement: S1 Fig — (TIF) [file pone.0154223.s001.tif]

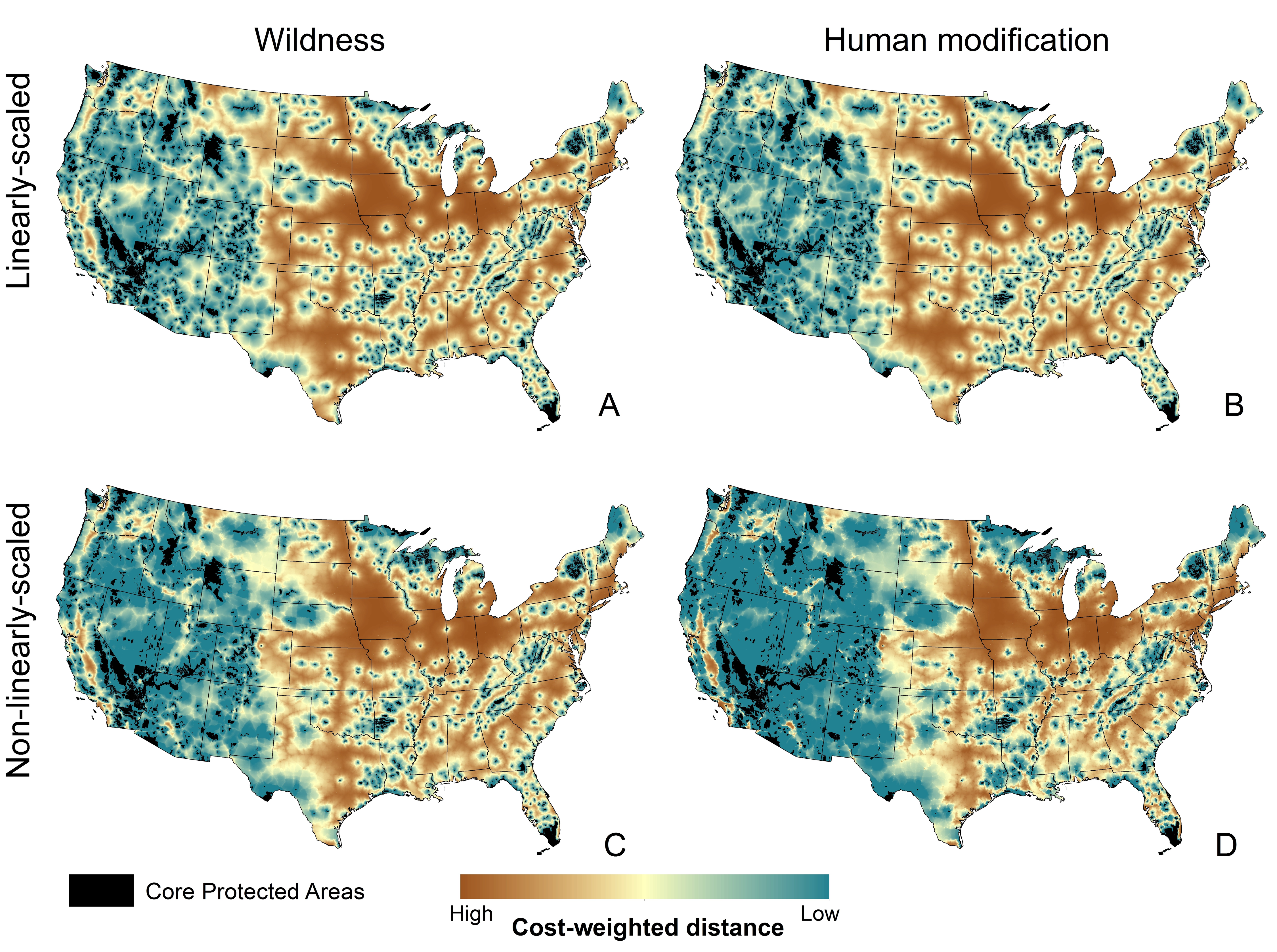

Supplement: S2 Fig — (TIF) [file pone.0154223.s002.tif]

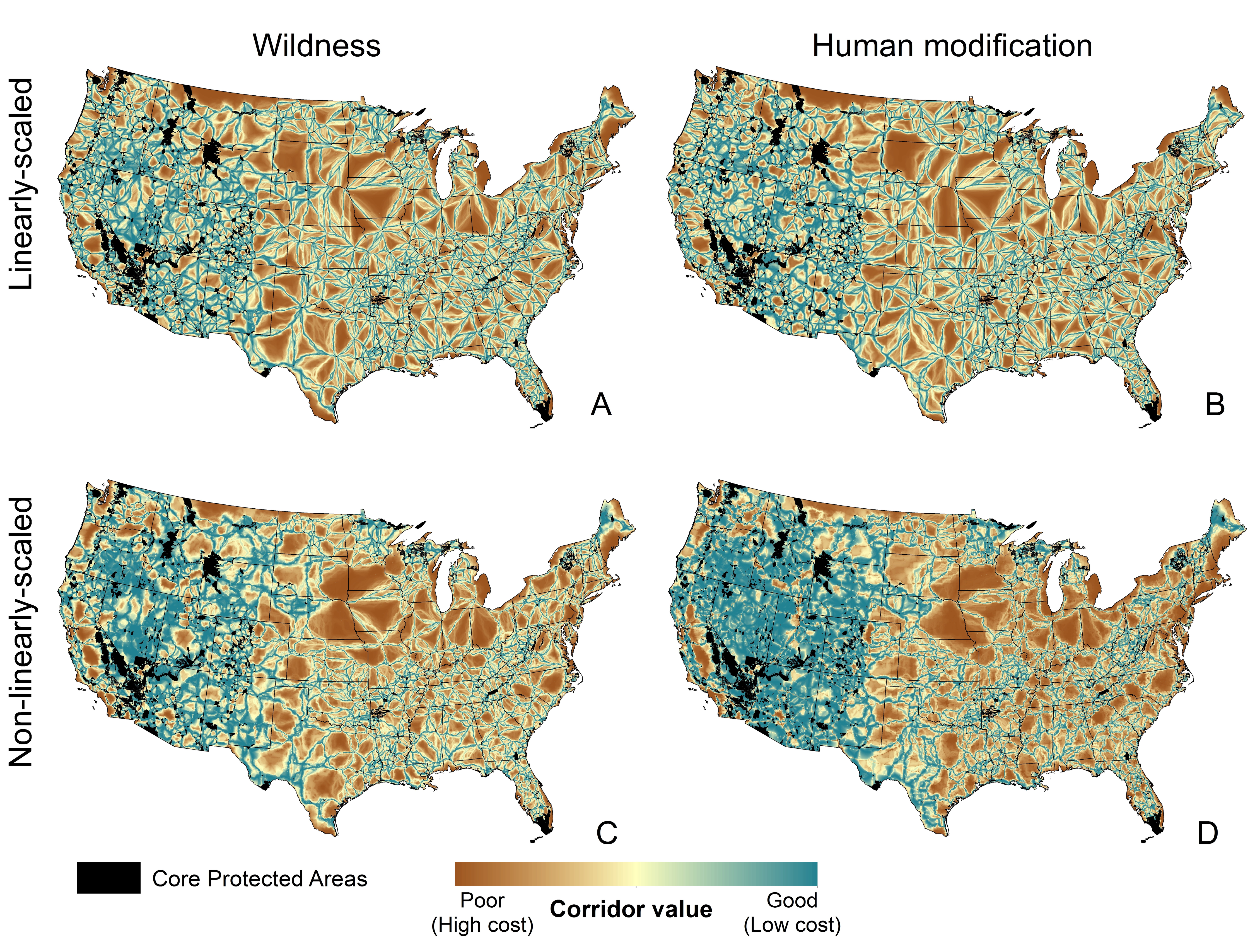

Supplement: S3 Fig — The mapped outputs shown here do not eliminate linkages between protected core areas based on any maximum geographic or cost-weighted distance, and only provided here as a reference of the most liberal modeled corridors. (TIF) [file pone.0154223.s003.tif]

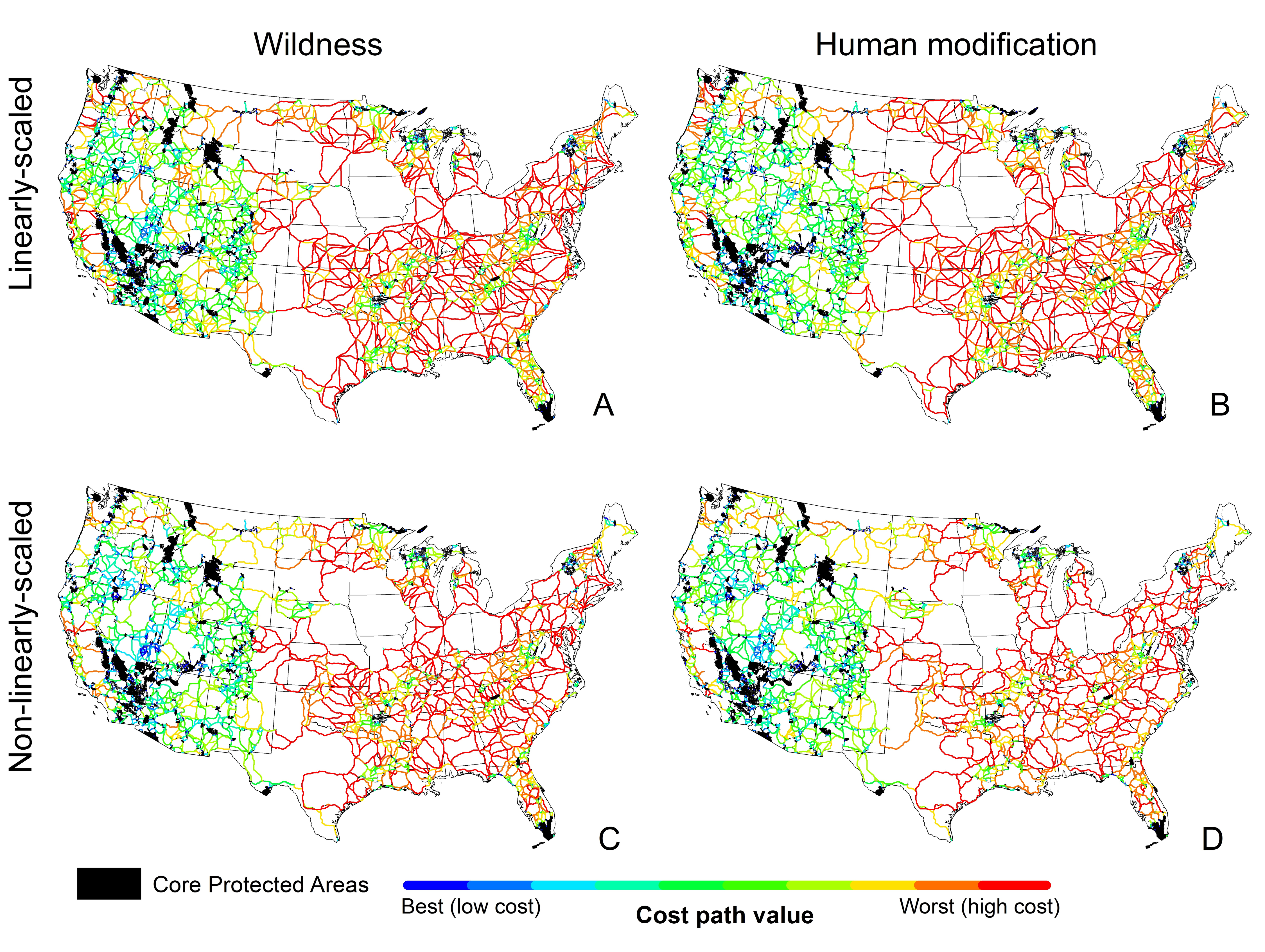

Supplement: S4 Fig — (TIF) [file pone.0154223.s004.tif]

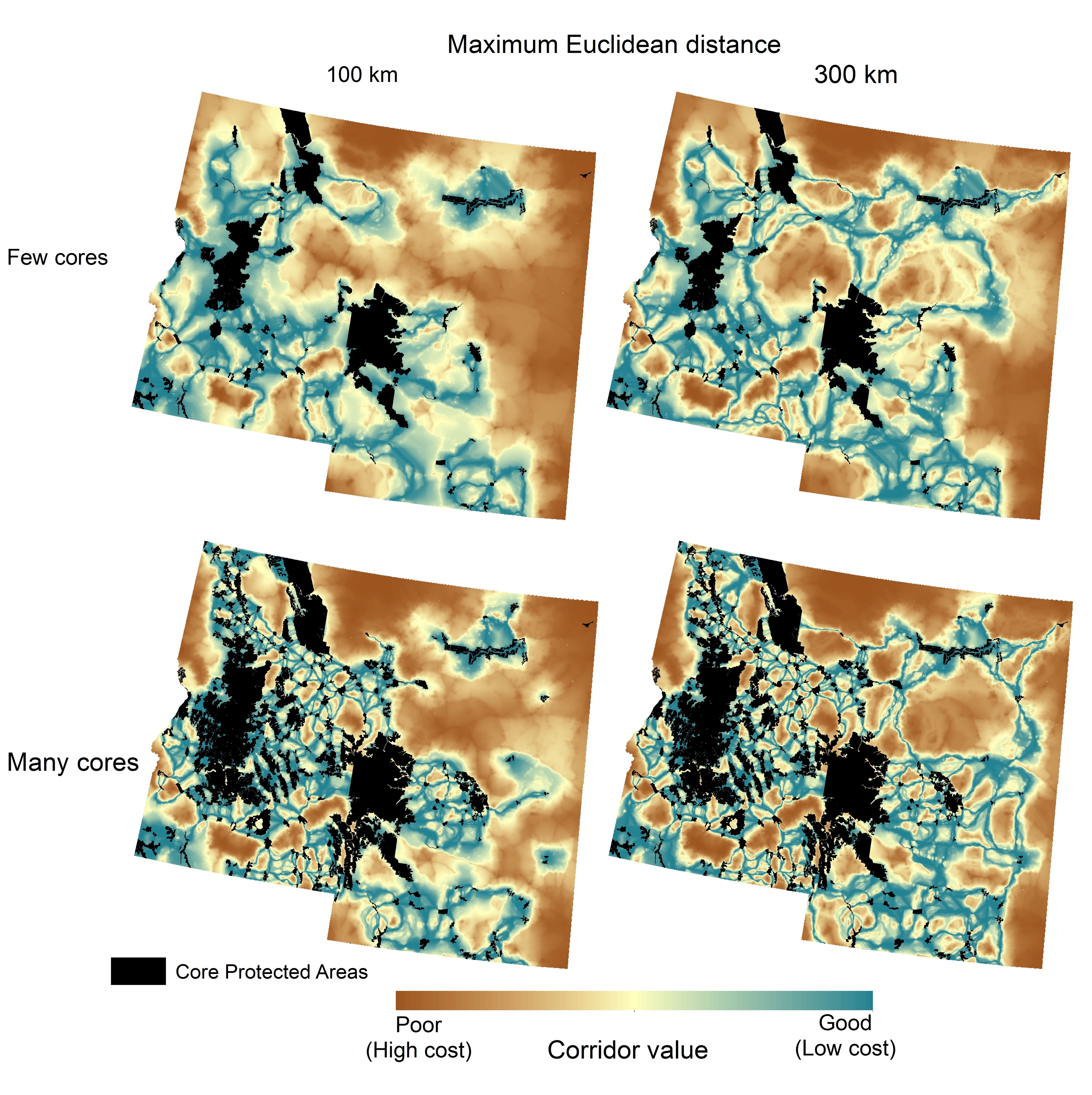

Supplement: S5 Fig — Here, we show a model experiment where we vary the maximum distance from 100 km to 300 km and cross this “treatment” with different number of cores (black polygons). The model runs with “few cores” included all of the large protected cores shown in Fig 1, while the model runs with “many cores” include all cores from Fig 1, plus all roadless and wilderness study area lands. Here, we demonstrate the sensitivity of Linkage Mapper to assumptions of core locations and maximum distances while demonstrating the importance that “stepping stones” may play in connectivity for a 3 state region (Idaho, Wyoming, and Montana). Connectivity that is limited by maximum dispersal distance between core areas may be overcome if additional smaller cores can serve as stepping stones between larger core areas. (TIF) [file pone.0154223.s005.tif]
