# Supplementary material for: Identifying Corridors among Large Protected Areas in the United States
Source: PLoS One. 2016 Apr 22;11(4):e0154223. doi: 10.1371/journal.pone.0154223 (PMC4841590; doi:10.1371/journal.pone.0154223)
Supplement: S1 Table — The number of units (N) is also reported. Agencies are rank ordered based on their overall mean corridor value so that agencies with the best corridor values are ranked higher. (DOCX) [file pone.0154223.s008.docx]

**S1 Table.** Mean and range of composite corridor values for federal, state, and local management agencies among units with varying GAP status values (1 = most protected; 4 = least protected). The number of units (N) is also reported. Agencies are rank ordered based on their overall mean corridor value so that agencies with the best corridor values are ranked higher.

|  | Gap status | | | | | | | | | | |  |  | | |  |
| --- | --- | --- | --- | --- | --- | --- | --- | --- | --- | --- | --- | --- | --- | --- | --- | --- |
|  | 1 | |  | 2 | |  | 3 | |  | 4 | |  | All | |  |  |
| Agency | Mean  (Range) | N |  | Mean  (Range) | N |  | Mean  (Range) | N |  | Mean (Range) | N |  | Mean (Range) | N |  |  |
| Land Trust | NA | 0 |  | NA | 0 |  | 35.8  (33.8-36.9) | 3 |  | NA | 0 |  | 35.8  (33.8-36.9) | 3 |  |  |
| Private University | NA | 0 |  | NA | 0 |  | 34.7 | 1 |  | NA | 0 |  | 34.7 | 1 |  |  |
| BLM | 34.1 | 1 |  | 35.1  (13.4-38) | 326 |  | 33.4  (15.1-38) | 679 |  | 30.7  (27-34.3) | 2 |  | 34  (13.4-38) | 1008 |  |  |
| DOE | NA | 0 |  | NA | 0 |  | NA | 0 |  | 33.4  (16.8-37.7) | 30 |  | 33.4  (16.8-37.7) | 30 |  |  |
| USFS | 33.1 | 1 |  | 32.4  (19.2-38) | 30 |  | 33.1  (4-38) | 1555 |  | 19.6  (8.3-31) | 2 |  | 33.1  (4-38) | 1588 |  |  |
| NRCS | NA | 0 |  | NA | 0 |  | NA | 0 |  | 32.7 | 1 |  | 32.7 | 1 |  |  |
| TNC | 33.8  (29.7-37.9) | 2 |  | 31.3  (19-37.3) | 4 |  | 32.5  (28.6-36.7) | 7 |  | 36.8 | 1 |  | 32.7  (19-37.9) | 14 |  |  |
| Regional Agency Land | NA | 0 |  | NA | 0 |  | 29.7  (16.1-36.9) | 3 |  | 32.3  (30.2-33) | 5 |  | 31.3  (16.1-36.9) | 8 |  |  |
| Other State Land | 19 | 1 |  | 31  (18.3-35.5) | 8 |  | 31.4  (4-37.7) | 72 |  | 29.7  (29.4-30) | 3 |  | 31.1  (4-37.7) | 84 |  |  |
| Unknown | NA | 0 |  | 35.1 | 1 |  | 30.6  (5-37.8) | 84 |  | 31.5  (14-38) | 72 |  | 31  (5-38) | 157 |  |  |
| County Land | NA | 0 |  | 35.6 | 1 |  | 30.3  (23.6-36.6) | 5 |  | 29.8  (28.2-31.4) | 2 |  | 30.8  (23.6-36.6) | 8 |  |  |
| Private Landowner | NA | 0 |  | 32.1  (28.1-37.3) | 6 |  | 32.2  (13.1-37.9) | 49 |  | 29.7  (10.9-37.7) | 105 |  | 30.5  (10.9-37.9) | 160 |  |  |
| State Land Board | NA | 0 |  | 26.7  (20-37.3) | 8 |  | 25.9  (10.7-37.9) | 72 |  | 31.8  (17-38) | 237 |  | 30.3  (10.7-38) | 317 |  |  |
| City Land | NA | 0 |  | NA | 0 |  | 31.1  (17.6-36) | 10 |  | 26.3  (25.7-26.9) | 2 |  | 30.3  (17.6-36) | 12 |  |  |
| State DOL | NA | 0 |  | NA | 0 |  | 30.2  (25.1-35.7) | 6 |  | NA | 0 |  | 30.2  (25.1-35.7) | 6 |  |  |
| Regional Water Districts | NA | 0 |  | 24 | 1 |  | 30.1  (14.4-37) | 54 |  | NA | 0 |  | 30  (14.4-37) | 55 |  |  |
| Native American Land | NA | 0 |  | NA | 0 |  | NA | 0 |  | 29.9  (5-37.9) | 156 |  | 29.9  (5-37.9) | 156 |  |  |
| Private Non-profit | NA | 0 |  | 32.6  (30.9-34.3) | 2 |  | 27.5  (7.2-35.7) | 6 |  | 30.9  (22-35.7) | 4 |  | 29.5  (7.2-35.7) | 12 |  |  |
| BOR | NA | 0 |  | NA | 0 |  | 28.9  (10-37.7) | 53 |  | 32.6 (27.2-36.9) | 3 |  | 29.1  (10-37.7) | 56 |  |  |
| Private Institution | 16.7  (7-26.3) | 2 |  | 14.7 | 1 |  | 28.7  (23.5-35.6) | 9 |  | 33.5  (28.2-37.4) | 7 |  | 28.5  (7-37.4) | 19 |  |  |
| State Park & Rec. | NA | 0 |  | 23.7  (8.8-36.1) | 6 |  | 29  (9.1-37.7) | 43 |  | NA | 0 |  | 28.3  (8.8-37.7) | 49 |  |  |
| BIA | NA | 0 |  | NA | 0 |  | NA | 0 |  | 28  (11.1-35.4) | 24 |  | 28  (11.1-35.4) | 24 |  |  |
| Joint Ownership | NA | 0 |  | 25.2  (17-34.5) | 4 |  | 30.1  (19-36) | 5 |  | 26.9 | 1 |  | 27.8  (17-36) | 10 |  |  |
| State Fish and Wildlife | 28.1 | 1 |  | 28.4  (9.1-36.8) | 43 |  | 27.2  (9.4-37.6) | 115 |  | 32.9  (26.8-35.9) | 6 |  | 27.7  (9.1-37.6) | 165 |  |  |
| State DNR | 34.3  (29.8-36.8) | 5 |  | 31.2  (12.9-37) | 10 |  | 27.2  (4-37.7) | 179 |  | 19.4 | 1 |  | 27.5  (4-37.7) | 195 |  |  |
| State DOC | NA | 0 |  | 27  (11-34.3) | 6 |  | 27.3  (4-37.9) | 37 |  | NA | 0 |  | 27.3  (4-37.9) | 43 |  |  |
| Other Federal Land | NA | 0 |  | 30.8 | 1 |  | 23.6  (17-30.2) | 2 |  | NA | 0 |  | 26  (17-30.8) | 3 |  |  |
| TVA | NA | 0 |  | 21.1 | 1 |  | 26.6  (19.5-31.3) | 6 |  | NA | 0 |  | 25.8  (19.5-31.3) | 7 |  |  |
| State University | NA | 0 |  | NA | 0 |  | 24.9 | 1 |  | NA | 0 |  | 24.9 | 1 |  |  |
| DOD | NA | 0 |  | NA | 0 |  | 27.9  (13-36.6) | 7 |  | 24.2  (4-38) | 304 |  | 24.3  (4-38) | 311 |  |  |
| ARS | NA | 0 |  | NA | 0 |  | 29.1  (21-33.6) | 3 |  | 17  (9-24.9) | 2 |  | 24.2  (9-33.6) | 5 |  |  |
| FWS | 18.6  (4-33.3) | 2 |  | 23.8  (4-37) | 46 |  | 24.2  (18-33) | 3 |  | NA | 0 |  | 23.6  (4-37) | 51 |  |  |
| NPS | 32.6  (28.2-36.2) | 3 |  | 23.4  (4-35.2) | 14 |  | 24.8  (12.5-34.5) | 13 |  | 13.7  (4-35.4) | 4 |  | 23.6  (4-36.2) | 34 |  |  |
| State Coastal Reserve | NA | 0 |  | 21  (20.3-21.8) | 2 |  | 18.3 | 1 |  | NA | 0 |  | 20.1  (18.3-21.8) | 3 |  |  |
| State DOE | 18.7 | 1 |  | 18.9  (18-19.8) | 2 |  | 20.2  (10.9-34.3) | 9 |  | NA | 0 |  | 19.8  (10.9-34.3) | 12 |  |  |
| State DOA | NA | 0 |  | NA | 0 |  | 18.9  (13.4-30.3) | 4 |  | NA | 0 |  | 18.9  (13.4-30.3) | 4 |  |  |
| State Cultural Affairs | NA | 0 |  | 17 | 1 |  | NA | 0 |  | NA | 0 |  | 17 | 1 |  |  |
| NOAA | NA | 0 |  | NA | 0 |  | 12.3  (4-31.9) | 17 |  | NA | 0 |  | 12.3  (4-31.9) | 17 |  |  |
| All | 28.4  (4-37.9) | 19 |  | 32.2  (4-38) | 524 |  | 31.8  (4-38) | 3113 |  | 28.7  (4-38) | 974 |  | 31.2  (4-38) | 4630 |  |  |
